# Bivariate Correlation Analysis of the Chemometric Profiles of Chinese Wild *Salvia miltiorrhiza* Based on UPLC-Qqq-MS and Antioxidant Activities

**DOI:** 10.3390/molecules23030538

**Published:** 2018-02-28

**Authors:** Xiaodan Zhang, Yange Yu, Yesheng Cen, Dongfeng Yang, Zhechen Qi, Zhuoni Hou, Shuanglai Han, Zengxuan Cai, Kuancheng Liu

**Affiliations:** 1 College of Life Science, Zhejiang Sci-Tech University, Hangzhou 310018, China; zxd_211@aliyun.com (X.Z.); cenyesheng@iCloud.com (Y.C.); ydf807@sina.com (D.Y.); zqi@zstu.edu.cn (Z.Q.); houzhuoni@zstu.edu.cn (Z.H.); 2 Institute of Soil and Water Conservation, Chinese Academy of Sciences & Ministry of Water Resources, Yangling 712100, China; 3 Industrial Crops Research Institute, Henan Academy of Agricultural Sciences, Zhengzhou 450002, China; yyg604208@163.com; 4 Department of Research and Development, Focused Photonics Inc., Hangzhou 310018, China; shuanglai_han@fpi-inc.com; 5 Department of Physicochemical and Toxicology, Zhejiang Provincial Center for Disease Control and Prevention, Hangzhou 310018, China; caizx_cdc@163.com

**Keywords:** *Salvia miltiorrhiza*, antioxidant activities, UPLC-Qqq-MS/MS, bivariate correlation analysis

## Abstract

To better understand the mechanisms underlying the pharmacological actions of *Salvia miltiorrhiza*, correlation between the chemical profiles and in vitro antioxidant activities in 50 batches of wild *S. miltiorrhiza* samples was analyzed. Our ultra-performance liquid chromatography–tandem mass spectrometry analysis detected twelve phenolic acids and five tanshinones and obtained various chemical profiles from different origins. In a principal component analysis (PCA) and cluster analysis, the tanshinones cryptotanshinone, tanshinone IIA and dihydrotanshinone I exhibited higher weights in PC1, whereas the phenolic acids danshensu, salvianolic acids A and B and lithospermic acid were highly loaded in PC2. All components could be optimized as markers of different locations and might be suitable for *S. miltiorrhiza* quality analyses. Additionally, the DPPH and ABTS assays used to comprehensively evaluate antioxidant activities indicated large variations, with mean DPPH and ABTS scavenging potencies of 32.24 and 23.39 μg/mL, respectively, among *S. miltiorrhiza* extract solutions. Notably, samples that exceeded the mean IC_50_ values had higher phenolic acid contents. A correlation analysis indicated a strong correlation between the antioxidant activities and phenolic acid contents. Caffeic acid, danshensu, rosmarinic acid, lithospermic acid and salvianolic acid B were major contributors to antioxidant activity. In conclusion, phenolic compounds were the predominant antioxidant components in the investigated plant species. These plants may be sources of potent natural antioxidants and beneficial chemopreventive agents.

## 1. Introduction

Many human disorders, such as coronary heart disease, inflammation, diabetes, carcinogenesis and neurodegenerative diseases, may result from increased concentrations of free radicals produced by numerous physiological and biochemical processes within the body [1,2]. Plants (vegetables, fruits, medicinal herbs) contain a wide variety of free radical-scavenging molecules, such as phenolic acids, flavonoids, quinones, alkaloids and betalains, which are rich in antioxidant activity [3,4]. Given their natural origins, plant-derived antioxidants are more beneficial than synthetic varieties, which were found to have undesirable secondary effects [5].

In recent years, the therapeutic effects of traditional Chinese medicine (TCM) agents have increasingly attracted attention [6]. The TCM agent danshen, or the root of *Salvia miltiorrhiza* Bge., is broadly accepted as a clinical treatment agent for cardiovascular and cerebrovascular diseases, dysmenorrhea and liver cirrhosis [7]. Two classes of major active compounds, namely tanshinones and phenolic acids, have been identified in *S. miltiorrhiza*. Both classes exhibit strong antioxidative activity and therefore contribute to the clinical applications of danshen. Tanshinones, which mainly comprise tanshinone I, tanshinone IIA and cryptotanshinone, have been reported to improve blood circulation [8] and exert antitumor [9] and anti-inflammatory functions [10]. Meanwhile, salvianolic acids, of which danshensu, caffeic acid and salvianolic B are the main examples, exert strong vasodilatory [11], oxidative [12] and antitumor activities [13]. According to the report [14], some chemical constituents of danshen have strong antioxidant capacities, and the curative effect of this TCM agent is based on the synergistic effects of various active antioxidant components.

Given its high value as a medicinal agent, *S. miltiorrhiza* is widely cultivated in many places for industrial consumption [15,16]. However, the quality and yield of *S. miltiorrhiza* are uneven and unstable when grown in different places [17]. In *S. miltiorrhiza*, phenolic acids and tanshinones have similar pharmacological effects (e.g., antioxidant and antiapoptotic functions), and many studies have indicated that chemical components and antioxidant activities could be used to evaluate the quality of *S. miltiorrhiza* [18,19,20]. Wu et al. [18] found that the antioxidant capacities of *S. miltiorrhiza* strains obtained from different habitats were proportional to the concentrations of total phenolic acids, and suggested that the pharmacodynamic method was somewhat accurate and feasible for evaluating herbal quality. Duan [21] constructed a model based on HPLC and NIR fingerprinting to assess the antioxidant activity of *S. miltiorrhiza* var. *alba* and provide useful references for the quality assurance and evaluation of this varietal. Although wild-grown *S. miltiorrhiza* has traditionally been considered to yield better quality materials than cultivars, previous reports have not evaluated the quality of wild *S. miltiorrhiza*, including the chemical characteristics and correlations with antioxidant activities. Therefore, the quality control of wild-grown *S. miltiorrhiza* populations in China depends on the quantification of secondary metabolites, evaluation of antioxidant activities and determination of the bioactive compounds that contribute to these activities. These steps may provide a scientific basis from which the benefits of wild *S. miltiorrhiza* can be fully utilised.

In this study, 50 individual roots of *S. miltiorrhiza* were collected from across China. A reliable and applicable quality control method based on ultra-performance liquid chromatography coupled to a triple quadruple mass spectrometry (UPLC-Qqq-MS/MS) was used to monitor 17 effective components (12 phenolic acids and 5 tanshinones) in *S. miltiorrhiza*. As phenolic acids and other natural antioxidant compounds are widely used to significantly reduce oxidative damage, assays based on relatively stable free radical-scavenging agents such as 1,1-diphenyl-2-picrylhydrazyl (DPPH) and 2,2′-azino-bis(3-ethylbenzthiazoline-6-sulfonic acid) (ABTS) were used to evaluate the antioxidant activity of *S. miltiorrhiza*. Concomitantly, a multivariate statistical approach, PCA and bivariate correlation analysis were adopted to assist the interpretation of correlations between secondary metabolites and antioxidant activities. This study may not only improve the current understanding of the bioactive compounds that contribute to antioxidant activity, but may also provide useful references for quality evaluations of wild *S. miltiorrhiza*.

## 2. Results and Discussion

### 2.1. Quantification of Compounds Investigated in S. miltiorrhiza

The compounds in wild-grown *S. miltiorrhiza* samples collected from different locations were clearly identified using the developed UPLC-Qqq-MS method (according to Section 2.2), and are summarized in Table S1. The contents of the 17 investigated compounds varied among the tested *S. miltiorrhiza* samples, although the proportions of the constituents were quite similar among the samples.

In general, Salvianolic acid B (**9**) was the most abundant component, and it ranged in concentration from 22.57 to 46.63 mg/g. The concentration of salvianolic acid B met the standard required by the Pharmacopoeia of the People’s Republic of China [22], except for samples S5, S11 and S38, which had salvianolic acid B concentrations of 25.13, 22.57 and 21.12 mg/g, respectively. The next most plentiful compounds were rosmarinic acid (**7**), lithospermic acid (**8**) and danshensu (**1**). The contents of protocatechualdehyde (**3**), cinnamic acid (**11**), protocatechuic acid (**2**), caffeic acid (**4**), ferulic acid (**5**) and isoferulic acid (**6**) were relatively low. However, these microconstituents could still play an important role in the Radix *Salvia miltiorrhiza*. The content of tanshinones (tanshinone IIA and cryptotanshinones) differed among the samples. The total content of cryptotanshinone, tanshinone IIA and tanshinone I ranged from 0.27 to 19.97 mg/g. The highest concentration was in sample S13 (Changzhi, China) and the lowest was in sample S45 (Hangzhou, China). According to the recent edition of the Chinese Pharmacopoeia (2015) [14], the total content of tanshinone I, tanshinone IIA and cryptotanshinone should not be less than 0.25%. More than 20 samples were not up to this standard, and most of these samples were from the southern provinces of Jiangxi, Hunan and Zhejiang. The coefficients of variation revealed that compounds from different locations were moderately variable, except for salvianolic acid C, tanshinone IIA and miltirone. The tanshinones compounds, including tanshinone IIA and cryptotanshinone, showed a larger range of variation than the phenolic acids. These results agree with earlier findings on *S. miltiorrhiza* samples from natural populations [23]. It was nonetheless surprising to find that the tanshinone content in samples from the south of China, such as the Jiangxi, Hunan and Zhejiang provinces, was lower than samples from the north.

### 2.2. UPLC-MS/MS Chromatograms of S. miltiorrhiza

**Figure 1:**
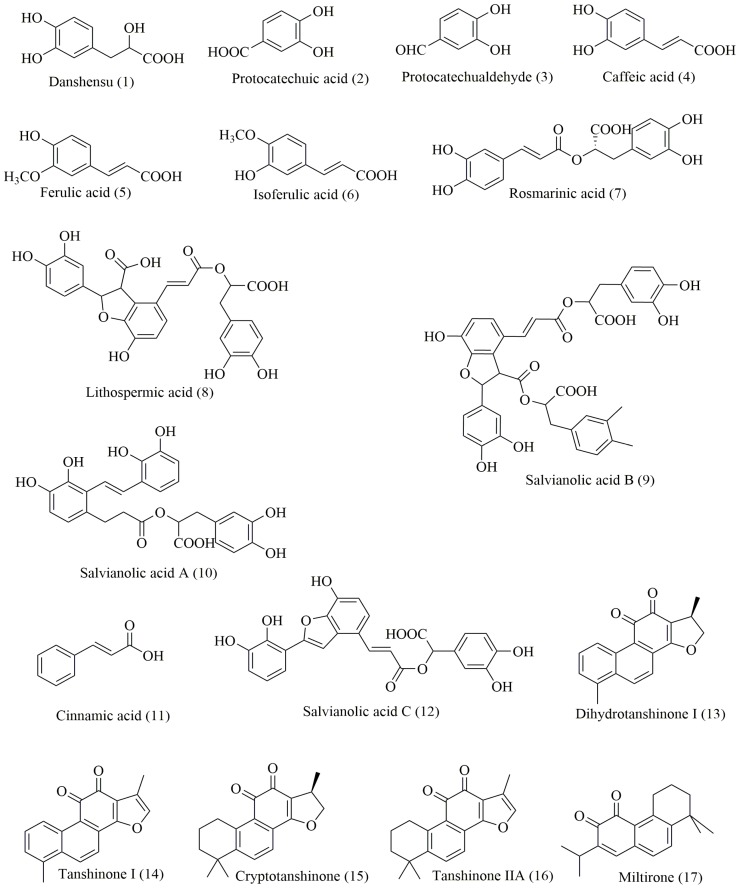
The structures of components identified in *Salvia miltiorrhiza*.

**Figure 2:**
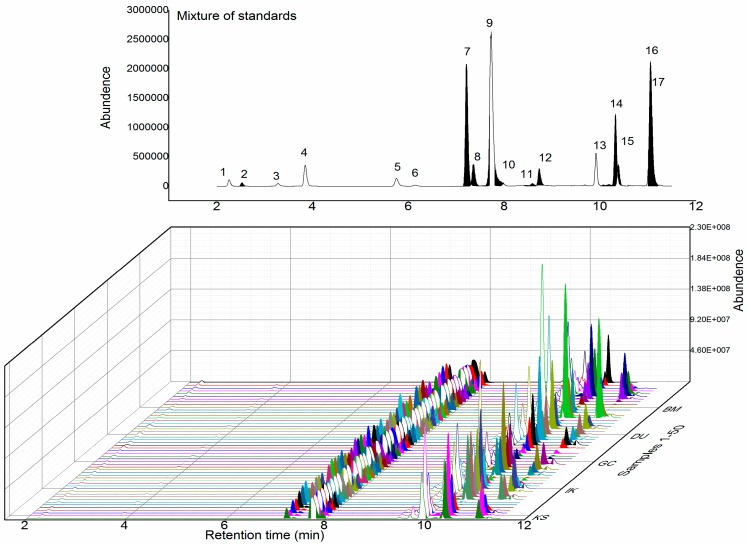
The ultra-performance liquid chromatography–tandem mass spectrometry chromatograms of a mixture of standards and *Salvia miltiorrhiza* samples. **1**. Danshensu; **2**. Protocatechuic acid; **3**. Protocatechualdehyde; **4**. Caffeic acid; **5**. Ferulic acid; **6**. Isferulic acid; **7**. Rosmarinic acid; **8**. Lithospermic acid; **9**. Salvianolic acid B; **10**. Salvianolic acid A; **11**. Cinnamic acid; **12**. Salvianolic acid C; **13**. Dihydrotanshinone I; **14**. Tanshinone I; **15**. Cryptotanshinone; **16**. Tanshinone IIA; **17**. Miltirone.

To assess the quality of 50 *S. miltiorrhiza* samples collected from wild locations around China, the profiles of tanshinones and phenolic acids, including danshensu (**1**), protocatechuic acid (**2**), protocatechualdehyde (**3**), caffeic acid (**4**), ferulic acid (**5**), isoferulic acid (**6**), rosmarinic acid (**7**), lithospermic acid (**8**), salvianolic acid B (**9**), salvianolic acid A (**10**), cinnamic acid (**11**), salvianolic acid C (**12**), dihydrotanshinone I (**13**), tanshinone I (**14**), cryptotanshinone (**15**), tanshinone IIA (**16**) and miltirone (**17**), were investigated (Figure 1). Figure 2 shows the UPLC-MS/MS chromatogram profiles of a standard mixture solution and 50 extracted samples. Compounds **1**–**17** were detected by UPLC-MS/MS, and the structures were further confirmed using exact mass data. The MS/MS fragmentation patterns and detailed information are shown in Table 1. The average relative retention times, average contents (*n* = 3) and coefficients of variance (C.V. %) of the contents of 17 common characteristic peaks are shown in Table S1. These C.V. % values all exceeded 12%, indicating that the content of each component varied significantly among different samples, and that the internal qualities differed among wild samples collected from different locations across China, but the proportions of the constituents were quite similar in each sample.

### 2.3. Antioxidant Capability Analysis

#### 2.3.1. Linear Relation Test

**Figure 3:**
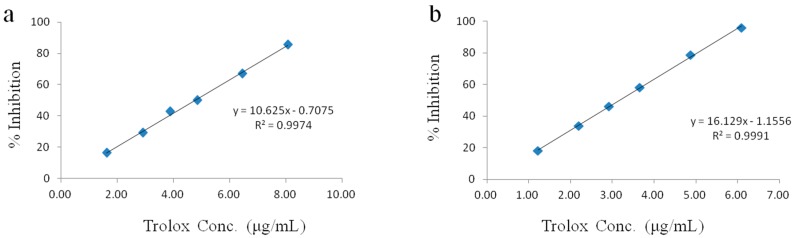
(**a**) Concentration-response curve for the absorbance at 517 nm of a standard Trolox solution in a 1,1-diphenyl-2-picrylhydrazyl (DPPH) assay; (**b**) Concentration–response curve for the absorbance at 734 nm of a standard Trolox solution in a 2,2′-azino-bis-(3-ethylbenzthiazoline-6-sulfonic acid) (ABTS) assay.

The antioxidant capabilities of different *S. miltiorrhiza* samples were determined using DPPH and ABTS assays, with Trolox as a positive control. Standard curves were obtained for both assays by measuring the ABTS and DPPH scavenging activities at different Trolox concentrations. The concentration-response curves for DPPH and ABTS are shown in Figure 3a,b, respectively. In the DPPH assay, the linear regression equation and coefficient of correlation for Trolox were *y* = 10.625*x* − 0.7075 and r^2^ = 0.9974, respectively, where y is the % inhibition and x is the Trolox concentration. In the ABTS assay, the corresponding results were *y* = 16.129*x* − 1.1556 and r^2^ = 0.9991, respectively.

#### 2.3.2. Evaluation of the Antioxidant Capacities of *S. miltiorrhiza* Extracts Using DPPH and ABTS Assays

The DPPH and ABTS scavenging effects of 50 samples of *S. miltiorrhiza* from different places were examined at different concentrations. The IC_50_ values shown in Table S2 indicate extremely large variations in the antioxidant activities of different *S. miltiorrhiza* samples. As with Trolox, the extract solutions exhibited antioxidant activity. As shown in Table S2, the r^2^ value for the efficiency curve of each sample exceeded 0.99, indicating a good relationship between the sample and the DPPH and ABTS inhibition rates. Here, a lower IC_50_ value implies higher antioxidant activity.

All samples exhibited strong, concentration-dependent antioxidant potentials against DPPH free radicals at 16.5449–85.2250 μg/mL. S8 most strongly inhibited DPPH (IC_50_ = 16.5449 μg/mL), whereas S11 exhibited the weakest inhibition (IC_50_ = 85.2250 μg/mL), a five-fold difference in antioxidant activity. The results of the ABTS assay paralleled those of the DPPH assay, with IC_50_ values of 12.7269–56.9827 μg/mL. The best ABTS free radical scavenging activities were exerted by samples S8 and S15 (IC_50_ = 12.7269 and 13.3907 μg/mL, respectively), whereas samples S38 and S11 exhibited lower activities (IC_50_ = 48.4268 and 56.9827 μg/mL, respectively). The *S. miltiorrhiza* extract solutions had mean DPPH and ABTS scavenging potencies of 32.2369 and 23.3933 μg/mL, respectively. A comparable evaluation of *S. miltiorrhiza* by Matkowski et al. yielded a mean DPPH IC_50_ value of 17.14 µg/mL [24], or slightly lower than the value obtained in this investigation. This discrepancy may be attributable to different extraction methods and sample collection locations.

Notably, samples that exceeded the mean IC_50_ values had higher phenolic acid contents. For instance, samples S8, S33 and S15 exhibited the strongest free radical-scavenging capabilities in both assays and had the highest total phenolic acid contents, whereas S11 and S38 possessed lower antioxidant capacities and lower phenolic acid contents. In addition, differences in altitude and environment influenced the sample contents. For example, the contents varied among four samples (S25, S26, S27, S28) from Ji’an (Jiangxi province), with maximum and minimum IC_50_ values of 33.8957 and 21.3639 μg/mL, respectively, in the DPPH assay. The antioxidant activities of these samples may have varied because they were collected from different habitats, such as wooded areas, fields, hillsides and roadsides.

### 2.4. PCA

A PCA was performed to evaluate the level of homogeneity in the quality of danshen collected from different locations in China. The PCA procedure facilitated the determination of the most important variables, or those representing a majority of the total information. As salvianolic acid C was not detected in some samples, only the contents of the other 16 investigated components were analyzed using SPSS. In this analysis, the first three principal components (F1–3) explained 63.888% of total variability among the 16 variables in the original data set (Table 2). The tanshinones cryptotanshinone, tanshinone IIA and dihydrotanshinone I were more heavily weighted in F1, whereas the phenolic acids danshensu, salvianolic acids A and B and lithospermic acid were highly loaded in F2. Thus, Cryptotanshinone, tanshinone IIA, dihydrotanshinone I, danshensu, salvianolic acids A and B and lithospermic acid could be optimised as location markers, and all might be suitable for evaluating the quality of danshen. Additionally, cryptotanshinone, tanshinone IIA and dihydrotanshinone I exhibited obvious positive phase loading in F1, indicating that the F1 increased with increasing concentrations of these tanshinones. The opposite finding was observed for danshensu and salvianolic acids A and B, consistent with earlier analyses of *S. miltiorrhiza* samples [25,26].

**Figure 4:**
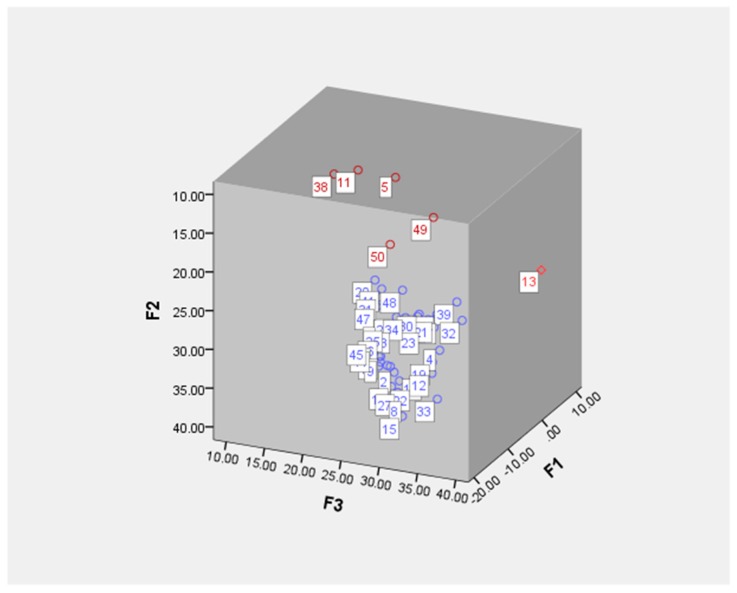
Loading plot generated from a principal component analysis (PCA) of wild *Salvia miltiorrhiza* samples collected from different locations.

An SPSS-based analysis of the investigated components in the PCA score plots (Figure 4) showed that S13 (Changzhi, China), which had the highest tanshinone concentration (28.4 mg/g), was distinct from the other samples. Although S5, S11, S38, S49 and S50, which had low total phenolic acid contents relative to the other samples, were considered to have clustered into one group, the remaining samples were clustered into another group, indicating that the proportions of the constituents were quite similar within this group. Overall, these results were similar to those obtained from the UPLC-Qqq-MS chemical profiles. To elucidate the relationships between the chemical profiles and antioxidant activities, a Pearson correlation analysis was subsequently introduced.

### 2.5. Correlation Analysis

A Pearson correlation analysis was used to evaluate the spectrum-effect relationships between the contents of 17 compounds and the IC_50_ values from the DPPH and ABTS free radical-scavenging assays. The results are shown in Table 3.

The correlation coefficients revealed close correlations of the antioxidant activities of methanolic *S. miltiorrhiza* extracts with the chemical compounds. The caffeic acid, danshensu, rosmarinic acid, lithospermic acid and salvianolic acid B, as well as total phenolic acids, exhibited obvious negative correlations with the IC_50_ values (*p* < 0.05). These negative correlations were most significant for salvianolic acid B and total phenolic acids (*p* < 0.01). A smaller IC_50_ value corresponds to stronger DPPH and ABTS free radical-scavenging abilities and, consequently, stronger antioxidant activity. The above results indicate that higher caffeic acid, danshensu, rosmarinic acid, lithospermic acid, salvianolic acid B and total phenolic acid contents corresponded to stronger antioxidant activities of *S. miltiorrhiza.* It was reported that the antioxidant capacity of the root of *Salvia miltiorrhiza* correlated with the total polyphenol and the hydroxycinnamic acid [24], including caffeic acid, danshensu, rosmarinic acid, lithospermic acid, salvianolic acid B etc. (such as the list in Figure 1). The results described herein highlight that the phenolic acid contents in *S. miltiorrhiza* were consistent with the assessed antioxidant tests, and that phenolic acids strongly contributed to the antioxidant activities of the *S. miltiorrhiza* samples analysed in this study. These findings were consistent with previous findings that the antioxidant capacity depends significantly on the contents of danshensu, salvianolic acid B and total phenolic acids [12,27]. Therefore, *S. miltiorrhiza* might be a source of potent natural antioxidants and beneficial chemo-preventive agents. By contrast, the result in this research indicated that the tanshinones content correlated positively with the IC_50_ values of DPPH and ABTS, corresponded to lower antioxidant activities. Specifically, dihydrotanshinone I exhibited a significant positive correlation with the IC_50_ values (*p* < 0.05) (as shown in Table 3). However, up to now, there are no reports providing evidence that tanshinones have a direct relationship with antioxidant activity. To find the reason, we calculated the intercorrelation coefficients between effective compounds shown in Table S4. It was indicated that tanshinones content correlated negatively with total phenolics, especially, dihydrotanshinone I exhibited a significant negative correlation with caffeic acid, rosmarinic acid, salvianolic acid B, and total phenolics, respectively. Thus, the general significant positive correlation of tanshinones with the IC_50_ values may be caused by the opposite content of phenolic acids in *S. miltiorrhiza* extracts, which were already proved to have strong antioxidant activities. In addition, a strong positive correlation was observed between the DPPH and ABTS assays, with a Pearson correlation coefficient of r = 0.980 (*p* < 0.01). This finding agrees with the results of the previous study, which determined a correlation coefficient of r = 0.949 between the DPPH and ABTS assays [28].

## 3. Materials and Methods

### 3.1. Materials and Chemicals

The *S. miltiorrhiza* samples used in this study were collected from wild locations around China. The plants were collected from 50 different geographic regions, including 12 provinces of China, at different altitudes (100–1200 m) from June to September 2015, as shown in Table S3. The samples were authenticated by Professor Yang Zongqi from the Department of Life Sciences at Zhejiang Sci-tech University according to the morphological characteristics of *S. miltiorrhiza*. Three replicates of representative plant samples were collected at each site. The plants were dried in an oven at 55 °C until they reached a constant weight.

Reference standards of tanshinone IIA, tanshinone I, cryptotanshinone, dihydrotanshinone I, ferulic acid, protocatechualdehyde and protocatechuic acid were provided by the Chinese Authenticating Institute of Material Medical and Biological Products (Beijing, China). Danshensu, caffeic acid, isoferulic acid, rosmarinic acid, salvianolic acid A, salvianolic acid B, salvianolic acid C, cinnamic acid, lithospermic acid and miltirone were purchased from Beijing Zhike Jiahua Instrument Company (Beijing, China). 1,1-diphenyl-2-picrylhydrazyl (DPPH), 2,2′-azino-bis-3-ethylbenzthiazoline-6-sulphonic acid (ABTS) and 6-hydroxy-2,5,7,8-tetramethylchroman-2-carboxilic acid (Trolox) were purchased from Hangzhou Maiyi Biological Technology Co., Ltd. (Hangzhou, China).

High-performance liquid chromatography (HPLC)-grade acetonitrile and formic acid were obtained from E. Merck (Darnstadt, Germany). Other chemicals and solvents were of analytical grade. Deionised water was purified using a Milli-Q system (Millipore, Bedford, MA, USA).

### 3.2. Determination of 17 Active Ingredients in Danshen

#### 3.2.1. Chromatographic Conditions

The UPLC system (Waters Corporation, Milford, MA, USA) was used for the analysis. Chromatographic separation was achieved on a Welch Ultimate XB-C18 column (2.1 mm × 100 mm, 1.8 μm, Welch Material Inc., Shanghai, China). The gradient elution system comprised (A) 0.1% formic acid and (B) acetonitrile as the mobile phase. For elution, the following gradient was applied: 0–1 min, 5–10% B; 1–2.5 min, 10–15% B; 2.5–5 min, 15–20% B; 5–8 min, 20–40% B; 8–8.2 min, 40–80% B; 8.2–11 min, 80% B; 11–11.1 min, 80–100% B; 11.1–12 min, 100% B; 12–12.1 min, 100–5% B; 12.1–15 min, 5% B. The flow-rate was 0.3 mL/min and the sample injection volume was 5 μL. The column temperature was maintained at 35 °C.

Tandem mass spectrometry analysis was performed on a Waters Xevo TQ mass spectrometer with an Electro Spray ionization source (Waters, Milford, MA, USA). Masslynx software ver. 4.1 (Waters Corporation, Milford, MA, USA) was used for data acquisition and analysis. The ionization mode was negative for phenolic acids and positive for tanshinones. Using MRM (multiple reaction monitoring), we selected the optimal conditions for the effective compounds. The desolvation gas flow was set at 800 L/h (L/hour) at a temperature of 500 °C, and the cone flow was 50 L/h. For the positive source, the capillary and cone voltages were set at 3.0 kV and 34 V, respectively. For the negative source, the capillary and cone voltages were 2.35 kV and 28 V, respectively. The retention times, precursor-to-product ion pairs, cone voltages and collision energies of the 17 standard compounds in *S. miltiorrhiza* are listed in Table 1.

#### 3.2.2. Standard Solutions Preparation

To prepare standard stock solution, the 17 reference compounds were accurately weighed and transferred to 10 mL volumetric flasks to which 5% acetonitrile was added to the graduation mark. The solutions were stored at 4 °C prior to use.

#### 3.2.3. Sample Preparation

*S. miltiorrhiza* samples were prepared according to previously published methods [29,30]. The dried roots were pulverized and sieved through No. 60 mesh (<0.250 mm). Each 0.2-g sample was accurately weighed and extracted in 20 mL of 70% methanol by ultrasonication for 60 min at room temperature, followed by centrifugation at 9000 rpm for 10 min. The supernatant was filtered through a 0.22-μm membrane before injection.

### 3.3. Antioxidant Activity

#### 3.3.1. Preparation of Trolox Solution

Trolox was dissolved in methanol to a concentration of 1 mg/mL, and subsequently diluted to different concentrations (0.02, 0.05, 0.09, 0.12, 0.15, 0.20 mg/mL) to establish a standard calibration curve.

#### 3.3.2. DPPH Assay

The antioxidant activities of 50 batches of *S. miltiorrhiza* samples were evaluated using DPPH free radical scavenging activity assays as previously reported, with some modifications [19,31,32]. A fresh DPPH stock solution was prepared by dissolving 394.32 mg DPPH in methanol to an absorbance of 0.700 ± 0.02 at 517 nm.

To ensure that extracts of all *S. miltiorrhiza* samples could reduce the stable free radical DPPH to the yellow-coloured DPPH, each sample solution was diluted to six different concentrations (range: 0.1–2.5 mg/mL). Extract solutions (0.1 mL) were mixed with 3 mL of a freshly prepared DPPH solution (0.1 mM in methanol). The mixture was shaken vigorously and kept in the dark at room temperature for 30 min, until stable absorbance values at 517 nm were obtained. The ability to scavenge DPPH radicals was calculated as a percentage according to the following equation:

% Inhibition = (A0 − A1)/A0 × 100%(1)

where A1 and A0 are the absorbance values of the sample and blank, respectively. The IC_50_ values, or concentrations of samples required to scavenge 50% of the DPPH free radicals, were calculated using a nonlinear regression analysis. Trolox was used as a reference compound for evaluating free radical scavenging activities.

#### 3.3.3. ABTS Assay

The ABTS free radical scavenging activity assay was performed as described previously, with slight modifications [28,33]. In this assay, the ABTS^+^ radical cation is generated by reacting 7 mM ABTS and 2.45 mM potassium persulfate via incubation at room temperature in the dark for 12–16 h. The ABTS solution was diluted with methanol to an absorbance of 0.700 ± 0.020 at 734 nm.

Each sample solution was diluted to six concentrations (range: 0.1–2.5 mg/mL). Subsequently, 100 µL of each plant extract solution were mixed with 4 mL of ABTS solution. The reactive mixture was allowed to stand at room temperature for 6 min in the dark, after which the absorbance was recorded immediately at 734 nm. The percentage of inhibition of absorbance at 734 nm was calculated using Equation (1).

### 3.4. Data Analysis

All statistical analyses were performed using Excel (Microsoft Corp., Redmond, WA, USA) and SPSS 19.0 software packages (SPSS, Inc., Chicago, IL, USA).

## 4. Conclusions

In the present study, the chemical compounds and in vitro antioxidant activities were determined simultaneously in 50 batches of wild *S. miltiorrhiza* samples collected from different locations in China. Twelve phenolic acids and five tanshinones were detected and quantified to provide a comprehensive overview of the chemical composition of wild-grown danshen. Regarding this composition, a PCA indicated that crytotanshinone, tanshinone IIA and dihydrotanshinone I, danshensu, salvianolic acids A and B and lithospermic acid could be optimized as location markers. In addition, the antioxidant activities of plant extracts could be primarily attributed to phenolic compounds. In this study, the different antioxidant activities of danshen correlated with the main chemical compounds and antioxidant capacity relationships in danshen could provide a tool with which to evaluate differences in the internal qualities and antioxidant activities of wild *S. miltiorrhiza* samples, as well as a sound experimental foundation and model for related studies.

## Figures and Tables

**Table 1:** The retention time, MS/MS fragment ions, cone voltage and collision energy for the UPLC-MS/MS method.

Code	Compounds	Retention Time (min)	Precursor Ion (*m*/*z*)	Product Ion (*m*/*z*)	Cone Voltage (V)	Collision Energy (eV)
**1**	Danshensu	2.26	197 [M − H]^−^	**135**	**18**	**17**
**2**	Protocatechuic acid	2.52	153 [M − H]^−^	**109**	**19**	**13**
**3**	Protocatechualdehyde	3.27	137 [M + H]^−^	**109**	**22**	**15**
**4**	Caffeic acid	3.83	179 [M − H]^−^	**135**	**20**	**15**
**5**	Ferulic acid	5.74	193 [M − H]^−^	**178**	**19**	**12**
**6**	Isferulic acid	6.14	193 [M − H]^−^	**134**	**18**	**18**
**7**	Rosmarinic acid	7.21	359 [M − H]^−^	**161**	**21**	**18**
**8**	Lithospermic acid	7.36	537 [M − H]^−^	**295**	**15**	**17**
**9**	Salvianolic acid B	7.74	717 [M − H]^−^	**519**	**23**	**16**
**10**	Salvianolic acid A	8	493 [M − H]^−^	**295**	**23**	**20**
**11**	Cinnamic acid	8.62	147 [M − H]^−^	**103**	**18**	**11**
**12**	Salvianolic acid C	8.78	491 [M − H]^−^	**293**	**22**	**21**
**13**	Dihydrotanshinone I	9.92	279 [M + H]^+^	**261**	**33**	**17**
**14**	Tanshinone I	10.33	277 [M + H]^+^	**178**	**35**	**30**
**15**	Cryptotanshinone	10.39	297 [M + H]^+^	**279**	**37**	**21**
**16**	Tanshinone IIA	11.07	295 [M + H]^+^	**249**	**35**	**25**
**17**	Miltirone	11.26	283 [M + H]^+^	**265**	**34**	**20**

**Table 2:** Total variance explained and component matrixes for the investigated compounds in *S. miltiorrhiza.*

Compound	Principal Components					Total
	1	2	3	4	5	
Cryptotanshinone	0.939	0.119	−0.132	0.098	0.021	1.044
Tanshinone IIA	0.931	0.092	−0.084	0.077	0.159	1.175
Dihydrotanshinone I	0.908	0.021	−0.179	0.035	0.170	0.955
Tanshinone I	0.702	0.075	−0.298	0.110	0.279	0.869
Miltirone	0.700	0.055	0.000	0.204	−0.231	0.727
Danshensu	0.056	0.899	0.105	0.287	0.108	1.455
Salvianolic acid A	0.199	0.846	−0.257	0.226	0.152	1.166
Lithospermic acid	0.007	0.775	0.426	−0.282	0.031	0.957
Protocatechuic acid	0.185	0.609	−0.133	0.512	0.366	1.539
Rosmarinic acid	−0.299	−0.078	0.854	−0.128	−0.212	0.138
Salvianolic acid B	−0.053	0.818	0.768	−0.222	−0.212	1.099
Caffeic acid	−0.330	−0.136	0.656	0.291	0.344	0.826
Protocatechualdehyde	0.220	0.154	0.031	0.806	0.250	1.461
Isferulic acid	0.098	0.098	−0.120	0.747	−0.086	0.737
Ferulic acid	0.178	0.149	0.054	0.115	0.718	1.215
Cinnamic acid	0.010	0.088	−0.177	0.000	0.693	0.614
Explained variance (%)	33.896	18.533	11.459	7.699	6.686	78.274
Cumulative contributory ratio (%)	33.896	52.429	63.888	71.588	78.274	78.274

**Table 3:** Correlation analysis between effective components and antioxidant activity in *S. miltiorrhiza*.

Compounds	Pearson Correlation		Compounds	Pearson Corrrelation	
	DPPH	ABTS		DPPH	ABTS
Tanshinone I	0.294 *	0.259	Ferulic acid	0.128	0.100
Dihydrotanshinone I	0.309 *	0.280 *	Isferulic acid	0.104	0.091
Miltirone	0.018	0.051	Danshensu	−0.357 *	−0.380 **
Tanshinone IIA	0.163	0.129	Rosmarinic acid	−0.740 **	−0.733 **
Cryptotanshinone	0.187	0.168	Salvianolic acid C	−0.121	−0.151
Total tanshinones	0.229	0.203	Salvianolic acid A	−0.067	−0.106
Protocatechualdehyde	0.046	0.000	Lithospermic acid	−0.569 **	−0.560 **
Cinnamic acid	0.241	0.206	Salvianolic acid B	−0.905 **	−0.889 **
Protocatechuic acid	0.050	−0.022	Total phenolic acids	−0.915 **	−0.904 **
Caffeic acid	−0.335 *	−0.347 *	DPPH	1	0.980 **

* *p* < 0.05, ** *p* < 0.01.

## Supplementary Materials

The supplementary materials are available online.

## Abbreviations

PCA	Principal component analysis

## References

[1] Boulila A., Sanaa A., Salem I.B., Rokbeni N., Mrabet Y., Hosni K., Fernandez X. (2015). Antioxidant properties and phenolic variation in wild populations of *Marrubium vulgare* L. (Lamiaceae). Ind. Crop Prod..

[2] Cai Y., Luo Q., Sun M., Croke H. (2004). Antioxidant activity and phenolic compounds of 112 traditional Chinese medicinal plants associated with anticancer. Life Sci..

[3] Yesil-Celiktas O., Nartop P., Gurel A., Bedir E., Sukan F.V. (2007). Determination of phenolic content and antioxidant activity of extracts obtained from *Rosmarinus officinalis*’ calli. J. Plant Physiol..

[4] Li X., Wang T., Zhou B., Gao W.Y., Cao J.G., Huang L.Q. (2014). Chemical composition and antioxidant and anti-inflammatory potential of peels and flesh from 10 different pear varieties (*Pyrus* spp.). Food Chem..

[5] Zheng W., Wang S.Y. (2001). Antioxidant activity and phenolic compounds in selected herbs. J. Agric. Food Chem..

[6] Kong W.J., Zhao Y.L., Xiao X.H., Wang J.B., Bing H., Li Z.L., Jin C., Liu Y. (2009). Spectrum-effect relationships between ultra performance liquid chromatography fingerprints and anti-bacterial activities of *Rhizoma coptidis*. Anal. Chim. Acta.

[7] Shu T., Pang M., Rong L., Zhou W., Wang J., Liu C., Wang X. (2014). Effects of *Salvia miltiorrhiza* on neural differentiation of induced pluripotent stem cells. J. Ethnopharmacol..

[8] Zhou C., Hu T., Li X., Jiao S., Wu Z. (2015). Determination of tanshinone II A in *Salvia miltiorrhiza* Bunge extract and its effect on ischemia-reperfusion injury. Indian J. Tradit. Knowl..

[9] Liu J.J., Wu H.H., Chen T.H., Leung W., Liang Y.C. (2015). 15,16-Dihydrotanshinone I from the functional food *Salvia miltiorrhiza* exhibits anticancer activity in human HL-60 leukemia cells: In vitro and in vivo studies. Int. J. Mol. Sci..

[10] Zhou Z., Wang S.Q., Liu Y., Miao A.D. (2006). Cryptotanshinone inhibits endothelin-1 expression and stimulates nitric oxide production in human vascular endothelial cells. Biochim. Biophys. Acta (BBA) Gen. Subj..

[11] Zhou X., Chan S.W., Tseng H.L., Deng Y., Hoi P.M., Choi P.S., Or P.M.Y., Yang J.M., Lam F.F.Y., Lee S.M.Y. (2012). Danshensu is the major marker for the antioxidant and vasorelaxation effects of Danshen (*Salvia miltiorrhiza*) water-extracts produced by different heat water-extractions. Phytomed. Int. J. Phytother. Phytopharm..

[12] Yang T.L., Lin F.Y., Chen Y.H., Chiu J.J., Shiao M.S., Tsai C.S., Lin S.J., Chen Y.L. (2011). Salvianolic acid B inhibits low-density lipoprotein oxidation and neointimal hyperplasia in endothelium-denuded hypercholesterolaemic rabbits. J. Sci. Food Agric..

[13] Zhang L.J., Chen L., Lu Y., Wu J.M., Xu B., Sun Z.G., Zheng S.Z., Wang A.Y. (2010). Danshensu has anti-tumor activity in B16F10 melanoma by inhibiting angiogenesis and tumor cell invasion. Eur. J. Pharmacol..

[14] Zhang D., Duan X., Deng S., Nie L., Zang H. (2015). Fingerprint analysis, multi-component quantitation, and antioxidant activity for the quality evaluation of *Salvia miltiorrhiza* var. *alba* by high-performance liquid chromatography and chemometrics. J. Sep. Sci..

[15] He C.E., Lu L.L., Jin Y., Wei J.H., Christie P. (2013). Effects of nitrogen on root development and contents of bioactive compounds in *Salvia miltiorrhiza* Bunge. Crop Sci..

[16] Zhao Q., Song Z., Fang X., Pan Y., Guo L.L., Liu T., Wang J.H. (2016). Effect of genotype and environment on *Salvia miltiorrhiza* roots using LC/MS-based metabolomics. Molecules.

[17] Zhao Y.J., Chen S.B., Gao G.Y., Feng Y.X., Yang S.L., Xu L.Z., Du L.J., Lin J., Li M. (2004). Content of inorganic elements of *Salvia miltiorrhiza* root in different area and physicochemical properties of its growing soil. Chin. J. Exp. Tradit. Med. Formul..

[18] Wu H., Tang L., Yang H. (2009). Quality evaluation of *Salvia miltiorrhiza* Bge by antioxidant activity. Chin. J. Exp. Tradit. Med. Formul..

[19] Jiang Y.Y., Wang L., Zhang L., Wang T., Liu Y., Ding C.B., Yang R.W., Wang X.L., Zhou Y.H. (2014). Characterization, antioxidant and antitumor activities of polysaccharides from *Salvia miltiorrhiza* Bunge. Int. J. Biol. Macromol..

[20] Sun G., Gao Y., Sun J. (2013). Correlation between quantified HPLC fingerprint and its anti-oxidation activity of *Salviae miltiorrhizae* Radix Et Rhizoma. Cent. South Pharm..

[21] Duan X. (2014). Study on Quality Evaluation and Fingerprint-Effect Relationship of Salvia Miltiorrhiza Var. Alba by Chemometrics.

[22] Chinese Pharmacopoeia Commission (2015). Pharmacopoeia of the People’s Republic of China.

[23] He Y.L. (2007). Studies on Main Contents of Radix Salviae Miltiorrhizae and Relation on Environment Factors.

[24] Matkowski A., Zielińska S., Oszmiański J., Lamer-Zarawska E. (2008). Antioxidant activity of extracts from leaves and roots of *Salvia miltiorrhiza* Bunge, *S. przewalskii* Maxim. and *S. verticillata* L.. Bioresour. Technol..

[25] Tokalıoğlu Ş. (2012). Determination of trace elements in commonly consumed medicinal herbs by ICP-MS and multivariate analysis. Food Chem..

[26] Wei H., Liu S.X., Liu Y., Zhang T.J., Chen C.Q. (2011). Comprehensive quality evaluation of *Salvia miltiorrhizae* radix et rhizoma. Drug Eval. Res..

[27] Kim M.H., Kim S.I., Seo D.W., Ryu J.C., Choi H.Y. (2010). Antioxidant activity of *Salvia miltiorrhiza* Bunge, a novel foodstuff. Mol. Cell. Toxicol..

[28] Floegel A., Kim D.O., Chung S.J., Koo S.I., Chun O.K. (2011). Comparison of ABTS/DPPH assays to measure antioxidant capacity in popular antioxidant-rich US foods. J. Food Compos. Anal..

[29] Zhong G.X., Li P., Zeng L.J., Guan J., Li D.Q., Li S.P. (2009). Chemical characteristics of *Salvia miltiorrhiza* (Danshen) collected from different locations in China. J. Agric. Food Chem..

[30] Liu A.H., Lin Y.H., Yang M., Guo H., Guan S.H., Sun J.H., Guo D.A. (2007). Development of the fingerprints for the quality of the roots of *Salvia miltiorrhiza* and its related preparations by HPLC-DAD and LC–MS^n^. J. Chromatogr. B.

[31] Zhang Y., Li Q., Xing H., Lu X.F., Zhao L.S., Qu K.K., Bi K.S. (2013). Evaluation of antioxidant activity of ten compounds in different tea samples by means of an on-line HPLC–DPPH assay. Food Res. Int..

[32] Musa K.H., Abdullah A., Kuswandi B., Hidayat M.A. (2013). A novel high throughput method based on the DPPH dry reagent array for determination of antioxidant activity. Food Chem..

[33] Tawaha K., Alali F.Q., Gharaibeh M., Mohammad M., Elimat T.E. (2007). Antioxidant activity and total phenolic content of selected Jordanian plant species. Food Chem..

