# Supplementary material for: Bivariate Correlation Analysis of the Chemometric Profiles of Chinese Wild Salvia miltiorrhiza Based on UPLC-Qqq-MS and Antioxidant Activities
Source: Molecules. 2018 Feb 28;23(3):538. doi: 10.3390/molecules23030538 (PMC6017509; doi:10.3390/molecules23030538)
Supplement: Supplementary file 1 [file molecules-23-00538-s001.pdf]

*molecules*

Article

# Bivariate Correlation Analysis of the Chemometric Profiles of Chinese Wild *Salvia miltiorrhiza* Based on UPLC-Qqq-MS and Antioxidant Activities

**Xiaodan Zhang [1,2,†], Yange Yu [3,†], Yesheng Cen [1], Dongfeng Yang [1], Zhechen Qi [1], Zhuoni Hou [1], Shuanglai Han [4], Zengxuan Cai [5] and Kuancheng Liu [1,*]**

1 College of Life Science, Zhejiang Sci-Tech University, Hangzhou 310018, China; zxd_211@aliyun.com (X.Z.); cenyesheng@iCloud.com (Y.C.); ydf807@sina.com (D.Y.); zqi@zstu.edu.cn (Z.Q.); houzhuoni@zstu.edu.cn (Z.H.)
2 Institute of Soil and Water Conservation, Chinese Academy of Sciences & Ministry of Water Resources, Yangling 712100, China
3 Industrial Crops Research Institute, Henan Academy of Agricultural Sciences, Zhengzhou 450002, China; yyg604208@163.com
4 Department of Research and Development, Focused Photonics Inc., Hangzhou 310018, China; shuanglai_han@fpi-inc.com
5 Department of Physicochemical and Toxicology, Zhejiang Provincial Center for Disease Control and Prevention, Hangzhou 310018, China; caizx_cdc@163.com
* Correspondence: kchliu@126.com; Tel.: +86-0571-8684-3192
† These authors contributed equally to this work.

**Table S1. Chemical profiles of 50 wild *Salvia miltiorrhiza* varieties**

| Sample code | Investigated compounds | | | | | | | | | | | | | | | | | | |
|---|---|---|---|---|---|---|---|---|---|---|---|---|---|---|---|---|---|---|---|
| | Twelve phenolic acids (mg/g, DW) | | | | | | | | | | | | | Five tanshinones (mg/g, DW) | | | | | |
| | 1 | 2 | 3 | 4 | 5 | 6 | 7 | 8 | 9 | 10 | 11 | 12 | Total | 13 | 14 | 15 | 16 | 17 | Total |
| S1 | 1.79 | 0.017 | 0.047 | 0.064 | 0.003 | 0.009 | 5.43 | 3.31 | 39.04 | 0.28 | 0.029 | 0.093 | 50.12 | 1.40 | 1.20 | 2.85 | 2.02 | 0.64 | 8.11 |
| S2 | 1.07 | 0.013 | 0.016 | 0.150 | 0.003 | 0.004 | 7.63 | 3.55 | 40.49 | 0.12 | 0.028 | 0.062 | 53.13 | 0.53 | 0.30 | 0.75 | 0.28 | 0.13 | 1.99 |
| S3 | 1.19 | 0.009 | 0.014 | 0.071 | 0.002 | 0.001 | 5.41 | 2.08 | 39.05 | 0.14 | 0.035 | 0.047 | 48.05 | 1.28 | 0.97 | 1.12 | 0.77 | 0.10 | 4.23 |
| S4 | 1.65 | 0.020 | 0.038 | 0.077 | 0.003 | 0.019 | 5.78 | 3.62 | 42.36 | 0.30 | 0.041 | 0.131 | 54.04 | 1.77 | 1.73 | 3.15 | 2.16 | 0.80 | 9.62 |
| S5 | 0.49 | 0.011 | 0.027 | 0.039 | 0.002 | 0.014 | 2.02 | 1.50 | 25.13 | 0.17 | 0.032 | 0.012 | 29.45 | 3.52 | 2.04 | 4.91 | 3.79 | 0.46 | 14.73 |
| S6 | 0.66 | 0.004 | 0.004 | 0.046 | 0.001 | 0.004 | 5.50 | 3.06 | 38.51 | 0.13 | 0.029 | 0.031 | 47.98 | 1.77 | 2.13 | 2.34 | 2.58 | 0.01 | 8.83 |
| S7 | 0.64 | 0.002 | 0.002 | 0.066 | 0.001 | 0.002 | 5.13 | 3.79 | 36.11 | 0.10 | 0.032 | 0.048 | 45.94 | 0.82 | 0.39 | 0.64 | 0.30 | 0.09 | 2.23 |
| S8 | 0.50 | 0.003 | 0.012 | 0.114 | 0.001 | 0.013 | 8.66 | 2.11 | 45.76 | 0.09 | 0.028 | 0.000 | 57.30 | 0.32 | 0.26 | 0.65 | 0.21 | 0.08 | 1.54 |
| S9 | 0.74 | 0.007 | 0.009 | 0.110 | 0.003 | 0.003 | 7.06 | 3.26 | 39.16 | 0.11 | 0.028 | 0.049 | 50.53 | 0.17 | 0.18 | 0.12 | 0.13 | 0.01 | 0.61 |
| S10 | 1.05 | 0.004 | 0.004 | 0.055 | 0.002 | 0.001 | 8.20 | 4.15 | 40.93 | 0.13 | 0.027 | 0.094 | 54.64 | 0.16 | 0.14 | 0.21 | 0.12 | 0.03 | 0.65 |
| S11 | 0.41 | 0.015 | 0.024 | 0.034 | 0.002 | 0.004 | 1.19 | 1.10 | 22.57 | 0.08 | 0.036 | 0.008 | 25.48 | 4.13 | 1.30 | 2.56 | 1.65 | 0.17 | 9.81 |
| S12 | 1.18 | 0.009 | 0.019 | 0.031 | 0.001 | 0.005 | 6.62 | 3.00 | 44.66 | 0.12 | 0.027 | 0.014 | 55.69 | 1.48 | 0.84 | 2.40 | 1.37 | 0.77 | 6.86 |
| S13 | 0.82 | 0.015 | 0.024 | 0.043 | 0.003 | 0.006 | 5.48 | 3.18 | 42.25 | 0.20 | 0.029 | 0.023 | 52.08 | 7.49 | 1.45 | 9.82 | 8.70 | 0.95 | 28.40 |
| S14 | 0.65 | 0.002 | 0.003 | 0.039 | 0.002 | 0.005 | 5.99 | 3.01 | 37.85 | 0.12 | 0.028 | 0.030 | 47.72 | 0.10 | 0.14 | 0.06 | 0.10 | 0.01 | 0.41 |

| | | | | | | | | | | | | | | | | | | |
|---|---|---|---|---|---|---|---|---|---|---|---|---|---|---|---|---|---|---|
| S15 | 0.59 | 0.006 | 0.021 | 0.089 | 0.002 | 0.011 | 11.12 | 2.88 | 44.79 | 0.10 | 0.028 | 0.006 | 59.64 | 0.24 | 0.35 | 0.17 | 0.13 | 0.02 | 0.91 |
| S16 | 1.63 | 0.019 | 0.029 | 0.059 | 0.002 | 0.004 | 5.79 | 4.26 | 43.41 | 0.20 | 0.029 | 0.082 | 55.52 | 1.30 | 0.42 | 2.03 | 1.06 | 0.41 | 5.21 |
| S17 | 1.00 | 0.013 | 0.028 | 0.041 | 0.002 | 0.010 | 5.03 | 2.84 | 40.69 | 0.17 | 0.028 | 0.029 | 49.88 | 2.96 | 0.80 | 4.69 | 2.27 | 0.09 | 10.81 |
| S18 | 0.69 | 0.004 | 0.003 | 0.029 | 0.001 | 0.015 | 6.74 | 3.01 | 39.08 | 0.15 | 0.032 | 0.028 | 49.77 | 0.22 | 0.30 | 0.14 | 0.26 | 0.01 | 0.94 |
| S19 | 2.36 | 0.022 | 0.028 | 0.069 | 0.002 | 0.014 | 4.57 | 3.62 | 43.30 | 0.42 | 0.037 | 0.054 | 54.50 | 1.64 | 0.83 | 2.60 | 1.79 | 0.32 | 7.18 |
| S20 | 0.68 | 0.009 | 0.019 | 0.032 | 0.002 | 0.002 | 3.39 | 1.91 | 33.37 | 0.20 | 0.028 | 0.022 | 39.67 | 1.14 | 0.62 | 1.58 | 0.98 | 0.45 | 4.77 |
| S21 | 0.74 | 0.005 | 0.016 | 0.036 | 0.001 | 0.004 | 7.03 | 2.75 | 38.98 | 0.13 | 0.027 | 0.008 | 49.73 | 2.47 | 0.37 | 5.77 | 1.35 | 0.94 | 10.90 |
| S22 | 1.30 | 0.007 | 0.009 | 0.031 | 0.003 | 0.004 | 5.57 | 3.29 | 45.09 | 0.18 | 0.027 | 0.011 | 55.52 | 0.51 | 0.36 | 1.14 | 0.45 | 0.30 | 2.77 |
| S23 | 0.84 | 0.003 | 0.004 | 0.032 | 0.002 | 0.004 | 6.62 | 3.17 | 39.26 | 0.12 | 0.026 | 0.006 | 50.09 | 1.56 | 1.30 | 3.38 | 1.77 | 0.45 | 8.47 |
| S24 | 2.15 | 0.035 | 0.030 | 0.061 | 0.003 | 0.008 | 2.74 | 4.04 | 36.41 | 0.40 | 0.031 | 0.201 | 46.11 | 1.09 | 0.62 | 2.07 | 0.94 | 0.19 | 4.91 |
| S25 | 0.71 | 0.007 | 0.046 | 0.065 | 0.001 | 0.005 | 7.84 | 1.92 | 36.85 | 0.09 | 0.030 | 0.001 | 47.56 | 1.05 | 0.90 | 0.93 | 1.14 | 0.09 | 4.10 |
| S26 | 0.56 | 0.005 | 0.019 | 0.047 | 0.003 | 0.003 | 5.94 | 2.14 | 38.61 | 0.09 | 0.034 | 0.004 | 47.45 | 0.47 | 0.49 | 0.47 | 0.46 | 0.04 | 1.93 |
| S27 | 0.75 | 0.004 | 0.018 | 0.091 | 0.001 | 0.002 | 7.42 | 3.00 | 43.94 | 0.09 | 0.045 | 0.012 | 55.37 | 0.21 | 0.26 | 0.22 | 0.18 | 0.02 | 0.89 |
| S28 | 0.82 | 0.005 | 0.011 | 0.039 | 0.001 | 0.002 | 6.87 | 2.70 | 43.39 | 0.11 | 0.027 | 0.007 | 53.98 | 0.38 | 0.35 | 0.65 | 0.27 | 0.13 | 1.78 |
| S29 | 0.75 | 0.006 | 0.024 | 0.099 | 0.002 | 0.003 | 9.00 | 2.05 | 39.47 | 0.07 | 0.028 | 0.001 | 51.51 | 0.14 | 0.25 | 0.17 | 0.12 | 0.02 | 0.69 |
| S30 | 0.56 | 0.003 | 0.009 | 0.057 | 0.001 | 0.003 | 5.21 | 1.94 | 39.55 | 0.10 | 0.028 | 0.001 | 47.46 | 1.73 | 1.54 | 2.76 | 2.11 | 0.99 | 9.14 |
| S31 | 0.51 | 0.003 | 0.010 | 0.113 | 0.002 | 0.008 | 4.21 | 2.00 | 35.02 | 0.10 | 0.030 | 0.001 | 42.01 | 0.88 | 0.45 | 1.86 | 0.57 | 0.53 | 4.29 |
| S32 | 1.10 | 0.005 | 0.009 | 0.041 | 0.002 | 0.005 | 5.60 | 3.65 | 41.19 | 0.18 | 0.028 | 0.033 | 51.84 | 4.68 | 0.62 | 6.24 | 2.34 | 0.10 | 13.99 |

| | | | | | | | | | | | | | | | | | | | |
|---|---|---|---|---|---|---|---|---|---|---|---|---|---|---|---|---|---|---|---|
| S33 | 0.98 | 0.006 | 0.014 | 0.069 | 0.002 | 0.005 | 7.43 | 3.92 | 46.63 | 0.14 | 0.030 | 0.022 | 59.25 | 1.36 | 0.64 | 2.38 | 1.00 | 0.43 | 5.80 |
| S34 | 0.41 | 0.002 | 0.001 | 0.027 | 0.001 | 0.006 | 6.43 | 2.43 | 37.66 | 0.14 | 0.034 | 0.012 | 47.14 | 1.56 | 1.14 | 2.47 | 1.13 | 0.93 | 7.22 |
| S35 | 0.58 | 0.004 | 0.006 | 0.030 | 0.001 | 0.010 | 6.82 | 2.43 | 39.56 | 0.12 | 0.030 | 0.009 | 49.60 | 0.13 | 0.23 | 0.11 | 0.20 | 0.02 | 0.69 |
| S36 | 0.92 | 0.008 | 0.010 | 0.049 | 0.002 | 0.003 | 7.47 | 3.11 | 40.26 | 0.12 | 0.031 | 0.034 | 52.03 | 0.45 | 0.48 | 0.38 | 0.45 | 0.06 | 1.83 |
| S37 | 0.91 | 0.025 | 0.018 | 0.069 | 0.002 | 0.005 | 6.05 | 3.14 | 38.00 | 0.20 | 0.028 | 0.073 | 48.51 | 0.37 | 0.37 | 0.47 | 0.26 | 0.10 | 1.57 |
| S38 | 0.53 | 0.008 | 0.030 | 0.021 | 0.001 | 0.013 | 0.70 | 1.02 | 21.12 | 0.15 | 0.028 | 0.009 | 23.64 | 1.24 | 0.71 | 2.57 | 0.96 | 0.59 | 6.07 |
| S39 | 1.17 | 0.022 | 0.034 | 0.061 | 0.003 | 0.007 | 4.95 | 2.87 | 39.93 | 0.16 | 0.033 | 0.029 | 49.26 | 3.12 | 1.98 | 5.40 | 3.73 | 0.80 | 15.04 |
| S40 | 0.89 | 0.004 | 0.008 | 0.036 | 0.002 | 0.005 | 5.07 | 2.73 | 39.74 | 0.15 | 0.029 | 0.007 | 48.67 | 2.37 | 0.57 | 4.20 | 1.51 | 0.87 | 9.52 |
| S41 | 0.81 | 0.012 | 0.028 | 0.071 | 0.003 | 0.015 | 5.38 | 1.28 | 34.16 | 0.08 | 0.028 | 0.000 | 41.87 | 1.59 | 0.53 | 2.33 | 0.94 | 0.20 | 5.59 |
| S42 | 1.00 | 0.016 | 0.037 | 0.037 | 0.002 | 0.011 | 4.59 | 2.68 | 41.40 | 0.17 | 0.028 | 0.028 | 50.00 | 1.96 | 0.89 | 4.34 | 1.99 | 0.48 | 9.65 |
| S43 | 1.15 | 0.011 | 0.008 | 0.029 | 0.002 | 0.007 | 3.59 | 2.87 | 40.66 | 0.34 | 0.029 | 0.016 | 48.71 | 2.04 | 1.74 | 4.38 | 2.58 | 0.10 | 10.84 |
| S44 | 0.53 | 0.008 | 0.020 | 0.165 | 0.002 | 0.005 | 7.06 | 2.38 | 38.17 | 0.09 | 0.030 | 0.015 | 48.48 | 0.11 | 0.23 | 0.05 | 0.10 | 0.02 | 0.50 |
| S45 | 0.39 | 0.003 | 0.005 | 0.041 | 0.001 | 0.002 | 6.59 | 1.49 | 38.42 | 0.10 | 0.030 | 0.000 | 47.07 | 0.04 | 0.13 | 0.04 | 0.10 | 0.01 | 0.32 |
| S46 | 0.62 | 0.002 | 0.001 | 0.032 | 0.001 | 0.003 | 5.33 | 2.93 | 41.66 | 0.16 | 0.028 | 0.030 | 50.81 | 0.20 | 0.39 | 0.04 | 0.10 | 0.04 | 0.77 |
| S47 | 0.54 | 0.006 | 0.011 | 0.037 | 0.002 | 0.005 | 3.66 | 2.57 | 35.76 | 0.12 | 0.031 | 0.017 | 42.76 | 0.65 | 0.50 | 1.19 | 0.53 | 0.24 | 3.11 |
| S48 | 0.55 | 0.005 | 0.009 | 0.039 | 0.005 | 0.007 | 3.86 | 2.68 | 35.76 | 0.19 | 0.035 | 0.029 | 43.17 | 1.91 | 1.30 | 2.69 | 1.81 | 0.27 | 7.97 |
| S49 | 1.06 | 0.022 | 0.025 | 0.030 | 0.004 | 0.005 | 1.56 | 2.75 | 31.18 | 0.27 | 0.040 | 0.084 | 37.03 | 4.42 | 2.03 | 6.00 | 4.03 | 0.31 | 16.78 |
| S50 | 0.37 | 0.005 | 0.017 | 0.025 | 0.002 | 0.007 | 3.06 | 1.69 | 31.22 | 0.10 | 0.032 | 0.000 | 36.53 | 2.26 | 0.69 | 4.06 | 1.88 | 0.19 | 9.08 |

| | | | | | | | | | | | | | | | | | | | |
|---|---|---|---|---|---|---|---|---|---|---|---|---|---|---|---|---|---|---|---|
| CV(%) | 49.10 | 78.99 | 68.18 | 55.76 | 41.48 | 65.05 | 36.12 | 28.64 | 13.63 | 49.74 | 12.96 | 122.35 | 15.62 | 99.04 | 73.88 | 94.50 | 112.80 | 102.79 | 90.22 |

1. Danshensu; 2. Protocatechuic acid; 3. Protocatechualdehyde; 4. Caffeic acid; 5. Ferulic acid; 6. Isferulic acid; 7. Rosmarinic acid; 8. Lithospermic acid; 9. Salvianolic acid B; 10. Salvianolic acid A; 11. Cinnamic acid; 12. Salvianolic acid C; 13. Dihydrotanshinone I; 14. Tanshinone I; 15. Cryptotanshinone; 16. Tanshinone IIA ; 17. Miltirone.

**Table S2. Results of antioxidant capability in different places of *S. miltiorrhiza***

| Sample code | Regression eq | | $R^2$ | | $IC_{50}$ (μg/mL） | |
|---|---|---|---|---|---|---|
| | DPPH | ABTS | DPPH | ABTS | DPPH | ABTS |
| S1 | y = 1.739x + 1.0138 | y = 2.566x + 0.8915 | 0.9997 | 0.9993 | 28.1692 | 19.1382 |
| S2 | y = 1.819x + 1.5661 | y=2.136+12.731 | 0.9994 | 0.9927 | 26.6267 | 17.4480 |
| S3 | y = 1.588x + 2.5685 | y=2.172x+4.1808 | 0.9953 | 0.9955 | 29.8687 | 21.0954 |
| S4 | y = 1.889x + 4.1088 | y = 2.715x - 4.2156 | 0.9913 | 0.991 | 24.2939 | 19.9689 |
| S5 | y = 0.808x + 1.0621 | y=1.248x+1.5313 | 0.9991 | 0.9975 | 60.5667 | 38.8371 |
| S6 | y =1.571x + 2.253 | y=2.124x+0.8815 | 0.999 | 0.9967 | 30.3927 | 23.1255 |
| S7 | y = 1.289x + 1.4497 | y=1.587x-1.4348 | 0.9965 | 0.9973 | 37.6651 | 32.4101 |
| S8 | y = 2.858x + 2.7148 | y = 3.910x+0.2379 | 0.9946 | 0.9995 | 16.5449 | 12.7269 |
| S9 | y = 1.580x + 0.1297 | y = 2.269x+3.2957 | 0.9973 | 0.9962 | 31.5635 | 20.5836 |
| S10 | y = 1.908x - 0.0794 | y = 2.228x+8.2593 | 0.9938 | 0.9965 | 26.2471 | 18.7346 |
| S11 | y = 0.565x + 1.8479 | y = 0.832x + 2.5904 | 0.9965 | 0.9961 | 85.2250 | 56.9827 |
| S12 | y = 2.073x + 3.0204 | y = 2.578x + 2.3079 | 0.9986 | 0.9932 | 22.6626 | 18.4997 |
| S13 | y = 1.894x + 1.288 | y = 2.484x + 3.3326 | 0.9975 | 0.9976 | 25.7191 | 18.7872 |
| S14 | y = 1.557x + 1.621 | y = 1.933x - 0.8819 | 0.9943 | 0.997 | 31.0719 | 26.3228 |
| S15 | y = 2.454x + 3.0832 | y = 3.889x - 2.0765 | 0.9979 | 0.9975 | 19.1185 | 13.3907 |
| S16 | y = 2.642x - 2.182 | y = 3.135x + 1.8245 | 0.9956 | 0.9957 | 19.7509 | 15.3670 |
| S17 | y = 1.914x + 0.4717 | y =2.689x + 2.1914 | 0.9976 | 0.9968 | 25.8769 | 17.7793 |
| S18 | y = 1.394x - 0.5783 | y = 2.143x - 1.094 | 0.9997 | 0.9976 | 36.2829 | 23.8423 |
| S19 | y = 1.878x + 1.2885 | y = 3.037x - 7.7037 | 0.999 | 0.9988 | 25.9380 | 19.0002 |
| S20 | y = 1.518x + 1.4355 | y = 2.942x + 2.3213 | 0.9982 | 0.9966 | 31.9924 | 23.3490 |
| S21 | y = 1.586x + 2.6846 | y = 1.994x + 3.2512 | 0.9987 | 0.9964 | 29.8332 | 23.4447 |
| S22 | y = 2.368x + 0.5635 | y = 2.839x + 0.9522 | 0.9978 | 0.9965 | 20.8769 | 17.2764 |
| S23 | y = 1.839x - 1.8809 | y = 2.400x - 1.7102 | 0.9962 | 0.9978 | 28.2115 | 21.5459 |
| S24 | y = 1.634x - 1.8429 | y= 2.048x + 3.3345 | 0.9959 | 0.9975 | 31.7276 | 22.7859 |
| S25 | y = 2.277x + 1.3545 | y = 2.972x + 0.7943 | 0.9985 | 0.9989 | 21.3639 | 16.5564 |
| S26 | y = 1.506x + 1.3545 | y = 2.031x + 0.0105 | 0.9943 | 0.9956 | 33.8957 | 24.6132 |

| | | | | | | |
|---|---|---|---|---|---|---|
| S27 | y = 2.163x + 0.4673 | y = 2.064x + 7.8243 | 0.9997 | 0.9934 | 22.9000 | 16.1965 |
| S28 | y = 1.896x + 3.4849 | y = 2.954x + 0.3666 | 0.9952 | 0.999 | 24.5333 | 16.8021 |
| S29 | y = 1.900x + 2.8888 | y = 2.554x + 3.1689 | 0.9955 | 0.9985 | 24.7954 | 18.3364 |
| S30 | y = 1.489x + 1.6804 | y = 1.831x + 2.4852 | 0.9977 | 0.997 | 32.4510 | 25.9502 |
| S31 | y = 1.575x + 2.7806 | y = 2.012x + 3.6016 | 0.9998 | 0.9962 | 29.9806 | 23.0608 |
| S32 | y = 1.891x + 3.8279 | y = 2.344x + 5.1419 | 0.9929 | 0.9908 | 24.4168 | 19.1374 |
| S33 | y = 2.624x + 3.0601 | y = 3.236x + 2.916 | 0.9991 | 0.9997 | 17.8887 | 14.5501 |
| S34 | y = 1.554x + 1.6607 | y = 2.235x + 0.802 | 0.9992 | 0.9996 | 31.1064 | 22.0125 |
| S35 | y = 1.484x + 2.4322 | y = 1.931x + 2.8026 | 0.9953 | 0.9937 | 32.0538 | 24.4419 |
| S36 | y = 2.042x + 0.9879 | y = 2.584x + 3.7799 | 0.9985 | 0.991 | 24.0020 | 17.8870 |
| S37 | y = 1.807x + 0.303 | y = 2.548x + 4.7965 | 0.9992 | 0.994 | 27.5025 | 17.7408 |
| S38 | y = 0.689x + 2.7603 | y = 0.983x + 2.3965 | 0.9969 | 0.9943 | 68.5627 | 48.4268 |
| S39 | y = 1.569x + 4.6095 | y = 2.517x + 1.1579 | 0.9969 | 0.9999 | 28.9296 | 19.4049 |
| S40 | y = 1.571x - 0.9355 | y = 1.893x + 2.9754 | 0.9922 | 0.9952 | 32.4223 | 24.8413 |
| S41 | y = 1.581x - 1.8948 | y = 1.992x + 0.7091 | 0.9979 | 0.9991 | 32.8240 | 24.7444 |
| S42 | y = 1.930x + 0.4095 | y = 2.532x - 2.3259 | 0.998 | 0.9951 | 25.6946 | 20.6658 |
| S43 | y = 1.459x + 1.6408 | y = 2.247x - 1.0428 | 0.9975 | 0.9959 | 33.1454 | 22.7160 |
| S44 | y = 1.500x + 1.932 | y = 1.912x + 3.0423 | 0.993 | 0.9957 | 32.0453 | 24.5595 |
| S45 | y = 1.366x + 2.1022 | y = 2.009x - 0.7853 | 0.9953 | 0.9984 | 35.0643 | 25.2789 |
| S46 | y = 1.843x + 2.3915 | y = 2.512x + 2.5477 | 0.9978 | 0.997 | 25.8321 | 18.8902 |
| S47 | y = 1.164x + 2.2764 | y = 1.590x + 4.2234 | 0.9981 | 0.9922 | 40.9997 | 28.7903 |
| S48 | y = 1.083x + 1.0093 | y = 1.363x + 2.3316 | 0.9998 | 0.9936 | 45.2361 | 34.9731 |
| S49 | y = 0.931x + 2.5459 | y = 1.595x + 0.2166 | 0.9946 | 0.9988 | 50.9711 | 31.2122 |
| S50 | y = 0.754x - 0.5189 | y=1.121x - 0.9335 | 0.998 | 0.9953 | 67.0012 | 45.4358 |
| Trolox | y = 10.625x – 0.7075 | y = 16.129x -1.1556 | 0.9974 | 0.9991 | 4.7725 | 3.1717 |
| mean | | | | | 32.2369 | 23.3933 |

**Table S3.** ***S. miltiorrhiza*** **populations included in this study, their collection sites, habitat and geographical coordinates.**

| Sample code | Place of origin or Geographic region | Latitude(°N) | Longitude(°E) | Altitude (m) | Habitat |
|---|---|---|---|---|---|
| S1 | Hanzhong, Shaanxi | 33.75598 | 106.33001 | 517 | Pine forest |
| S2 | Guilin, Guangxi | 25.285235 | 110.275236 | 154 | Roadside |
| S3 | Guilin, Guangxi | 25.285825 | 110.276488 | 158 | Park |
| S4 | Shangluo, Shaanxi | 33.271065 | 110.354161 | 462 | Hillside |
| S5 | Lingbao, Henan | 34.195045 | 110.430931 | 703 | Roadside |
| S6 | Shiyan, Hubei | 32.016233 | 110.566046 | 715 | Field |
| S7 | Shiyan, Hubei | 32.016797 | 110.566581 | 689 | Hillside |
| S8 | Shiyan, Hubei | 32.03189 | 110.568964 | 544 | Mountain roads |
| S9 | Zhangjiajie, Hunan | 29.240994 | 110.724758 | 159 | Hillside |
| S10 | Zhangjiajie, Hunan | 29.240094 | 110.724758 | 149 | Hillside |
| S11 | Nanyang, Henan | 33.274778 | 111.533463 | 608 | Grass |
| S12 | Yuncheng, Shanxi | 35.279606 | 111.884369 | 537 | Hillside |
| S13 | Changzhi, Shanxi | 36.64011 | 112.318259 | 1217 | Valley |
| S14 | Hengyang, Hunan | 27.281111 | 112.729685 | 390 | Hill |
| S15 | Hengyang, Hunan | 27.281857 | 112.730637 | 353 | Hill |
| S16 | Jiaozuo, Henan | 35.337025 | 113.252312 | 382 | Grass |
| S17 | Yangquan, Shanxi | 38.205892 | 113.59612 | 1169 | Field |

| | | | | | |
|---|---|---|---|---|---|
| S18 | Tongcheng, Hubei | 29.316633 | 113.670079 | 132 | Behind a house |
| S19 | Anyang, Henan | 36.162731 | 113.718918 | 668 | Roadside |
| S20 | Anyang, Henan | 36.163708 | 113.719491 | 670 | Roadside |
| S21 | Xingtai, Hebei | 37.156893 | 113.94061 | 533 | Chestnut forest |
| S22 | Xingtai, Hebei | 37.157747 | 113.943564 | 553 | Woods |
| S23 | Xingtai, Hebei | 37.159808 | 113.943781 | 496 | Side of a village |
| S24 | Xinyang, Henan | 31.502254 | 114.01514 | 238 | Roadside |
| S25 | Ji'an, Jiangxi | 26.312302 | 114.114013 | 418 | Woods |
| S26 | Ji'an, Jiangxi | 26.311784 | 114.114088 | 407 | Grass |
| S27 | Ji'an, Jiangxi | 26.31209 | 114.11409 | 415 | Grass |
| S28 | Ji'an, Jiangxi | 26.312522 | 114.114259 | 429 | Brookside |
| S29 | Ganzhou, Jiangxi | 25.382957 | 114.180527 | 816 | Bamboo forest |
| S30 | Wuhan, Hubei | 30.553826 | 114.414365 | 20 | Park |
| S31 | Baoding, Hebei | 39.271298 | 115.305802 | 143 | Hill |
| S32 | Baoding, Hebei | 39.269757 | 115.307681 | 152 | Roadside |
| S33 | Baoding, Hebei | 39.268056 | 115.308162 | 148 | Roadside |
| S34 | Huanggang, Hubei | 30.78971 | 115.367631 | 94 | Hill |
| S35 | Jiujiang, Jiangxi | 29.538842 | 116.031751 | 354 | Woods |
| S36 | Jiujiang, Jiangxi | 29.538866 | 116.031758 | 362 | Temple nearby |
| S37 | Nanfeng, Jiangxi | 27.035884 | 116.230774 | 165 | Valley |
| S38 | Jinan, Shandong | 36.32271 | 117.152962 | 452 | Grass |
| S39 | Linyi, Shandong | 35.145267 | 117.331005 | 352 | Hill |
| S40 | Tangshan, Hebei | 40.207318 | 117.665181 | 142 | Pine forest |
| S41 | Laiwu, Shandong | 36.353555 | 117.813792 | 353 | Hill |

| | | | | | |
|---|---|---|---|---|---|
| S42 | Laiwu, Shandong | 36.353827 | 117.813812 | 317 | Field |
| S43 | Laiwu, Shandong | 36.351476 | 117.818788 | 387 | Woods |
| S44 | Xuancheng, Anhui | 30.602898 | 118.696055 | 278 | Roadside |
| S45 | Hangzhou, Zhejiang | 29.791199 | 118.97704 | 480 | Hillside |
| S46 | Linan, Zhejiang | 29.361699 | 119.022079 | 350 | Roadside |
| S47 | Qinhuangdao, Hebei | 39.817158 | 119.450445 | 90 | Roadside |
| S48 | Qinhuangdao, Hebei | 39.819352 | 119.451303 | 65 | Bushes |
| S49 | Qingdao, Shandong | 36.160122 | 120.302143 | 187 | Pine trees |
| S50 | Dalian, Liaoning | 38.922769 | 121.502179 | 75 | Highway side |

**Table S4. Intercorrelations between effective compounds of S. miltiorrhiza.**

| | Tanshinone I | Dihydrotanshinone I | Miltirone | Tanshinone IIA | Cryptotanshinone | **Total tanshinones** | Protocatechualdehyde | Cinnamic acid | Protocatechuic acid | Caffeic acid | Ferulic acid | Isferulic acid | Danshensu | Rosmarinic acid | Salvianolic acid C | Salvianolic acid A | Lithospermic acid | Salvianolic acid B | **Total phenolics** |
|---|---|---|---|---|---|---|---|---|---|---|---|---|---|---|---|---|---|---|---|
| Tanshinone I | **1** | | | | | | | | | | | | | | | | | | |
| Dihydrotanshinone I | **0.635**** | **1** | | | | | | | | | | | | | | | | | |
| Miltirone | **0.449**** | **0.449**** | **1** | | | | | | | | | | | | | | | | |
| Tanshinone IIA | **0.732**** | **0.913**** | **0.530**** | **1** | | | | | | | | | | | | | | | |
| Cryptotanshinone | **0.634**** | **0.938**** | **0.620**** | **0.897**** | **1** | | | | | | | | | | | | | | |
| **Total tanshinones** | **0.738**** | **0.965**** | **0.614**** | **0.962**** | **0.974**** | **1** | | | | | | | | | | | | | |
| Protocatechualdehyde | **0.286** | **0.297** | **0.134** | **0.314*** | **0.226** | **0.303** | **1** | | | | | | | | | | | | |
| Cinnamic acid | **0.308** | **0.139** | **0.064** | **0.138** | **0.062** | **0.134** | **0.317*** | **1** | | | | | | | | | | | |
| Protocatechuic acid | **0.343*** | **0.326*** | **0.119** | **0.354*** | **0.315*** | **0.345*** | **0.652**** | **0.284** | **1** | | | | | | | | | | |
| Caffeic acid | **-0.327*** | **-0.370*** | **-0.201** | **-0.310*** | **-0.378*** | **-0.372*** | **0.239** | **0.042** | **0.027** | **1** | | | | | | | | | |
| Ferulic acid | **0.256** | **0.249** | **0.043** | **0.252** | **0.219** | **0.247** | **0.305** | **0.255** | **0.412**** | **0.006** | **1** | | | | | | | | |
| Isferulic acid | **0.243** | **0.142** | **0.128** | **0.179** | **0.185** | **0.189** | **0.428**** | **0.206** | **0.343**** | **0.134** | **0.162** | **1** | | | | | | | |
| Danshensu | **0.124** | **0.092** | **0.091** | **0.139** | **0.181** | **0.149** | **0.375*** | **0.203** | **0.700**** | **-0.011** | **0.199** | **0.316*** | **1** | | | | | | |

| | | | | | | | | | | | | | | | | | | | |
|---|---|---|---|---|---|---|---|---|---|---|---|---|---|---|---|---|---|---|---|
| Rosmarinic acid | -0.537** | -0.520** | -0.216 | -0.432** | -0.465** | -0.501** | -0.139 | -0.29 | -0.438** | 0.438** | -0.296 | -0.087 | -0.152 | 1 | | | | | |
| Salvianolic acid C | 0.107 | 0.013 | -0.03 | 0.048 | 0.026 | 0.036 | 0.235 | 0.259 | 0.691** | 0.020 | 0.245 | 0.189 | 0.691** | -0.232 | 1 | | | | |
| Salvianolic acid A | 0.360* | 0.261 | 0.16 | 0.334* | 0.343* | 0.337* | 0.256 | 0.262 | 0.719** | -0.211 | 0.251 | 0.348* | 0.832** | -0.445** | 0.677** | 1 | | | |
| Lithospermic acid | -0.088 | -0.053 | -0.033 | 0.012 | 0.031 | -0.01 | -0.178 | 0.034 | 0.213 | 0.006 | 0.045 | 0.045 | 0.619** | 0.22 | 0.615** | 0.473** | 1 | | |
| Salvianolic acid B | -0.331* | -0.309* | -0.016 | -0.167 | -0.168 | -0.227 | -0.181 | -0.172 | -0.177 | 0.235 | -0.194 | -0.01 | 0.316* | 0.700** | 0.008 | 0.082 | 0.580** | 1 | |
| **Total phenolics** | -0.383* | -0.363* | -0.07 | -0.227 | -0.233 | -0.291 | -0.155 | -0.181 | -0.17 | 0.292 | -0.199 | -0.01 | 0.318* | 0.786** | 0.062 | 0.046 | 0.622** | 0.983** | 1 |

* shows significance at the level as p=0.05; ** shows significance at the level as p=0.01
